# Characteristics and Outcomes of Cryptococcosis among Patients with and without COVID-19

**DOI:** 10.3390/jof8111234

**Published:** 2022-11-21

**Authors:** Daniel B. Chastain, Vanessa M. Kung, Lilian Vargas Barahona, Brittany T. Jackson, Sahand Golpayegany, Carlos Franco-Paredes, George R. Thompson, Andrés F. Henao-Martínez

**Affiliations:** 1 Department of Clinical and Administrative Pharmacy, University of Georgia College of Pharmacy, Albany, GA 31701, USA; 2 Division of Infectious Diseases, University of Colorado, Anschutz Medical Campus, Aurora, CO 80045, USA; 3 Department of Pharmacy, Mount Sinai Morningside, New York, NY 10025, USA; 4 Hospital Infantil de México, Federico Gómez, México City 06720, Mexico; 5 Department of Microbiology, Immunology, and Pathology, Colorado State University, Fort Collins, CO 80523, USA; 6 Department of Medicine, Division of Infectious Diseases, University of California, Davis Medical Center, Sacramento, CA 95616, USA

**Keywords:** SARS-CoV-2, COVID-19, immunotherapy, cryptococcus

## Abstract

The effect of COVID-19 on the risk and prognosis of cryptococcosis is unclear. We compared the characteristics and outcomes of cryptococcosis in patients with and without COVID-19. Patients 18 years and older with cryptococcosis were identified from TriNetX and separated into two cohorts based on a diagnosis of COVID-19 within 3 months of the index diagnosis of cryptococcosis. Differences examined between groups included comorbidities, immunosuppressive medications, ED visits, hospitalizations, ICU admissions, mechanical ventilation, and deaths. The propensity score matching was performed based on demographics and comorbidities. Of the 6998 patients with cryptococcosis included, 4.4% (*n* = 306) had COVID-19 prior to cryptococcosis. Mortality was higher in patients with COVID-19 compared to those without COVID-19 (14% vs. 11%, *p* = 0.032). Additionally, those with COVID-19 were older (55.2 ± 14.4 vs. 51.9 ± 15.2 years, *p* < 0.001) with higher rates of transplant (29% vs. 13%, *p* < 0.001), neoplastic disease (37% vs. 21%, *p* < 0.001), chronic kidney disease (42% vs. 18%, *p* < 0.001), or diabetes (35% vs. 19%, *p* < 0.001) but not HIV (30% vs. 31%, *p* = 0.618). Glucocorticoid use was more common in those with COVID-19 (52% vs. 27%, *p* < 0.001). More patients with COVID-19 required ED visits (29% vs. 23%, *p* = 0.025) and ICU admission (18% vs. 11%, *p* < 0.001). After propensity score matching, patients with COVID-19 had higher rates of neoplastic disease, heart failure, chronic kidney disease, and glucocorticoid use but did not experience worse outcomes compared to those without COVID-19. Patients with COVID-19 who developed cryptococcosis had independently higher rates of comorbidities and glucocorticoid use but similar outcomes, including death, versus those without COVID-19.

## 1. Introduction

Cryptococcosis primarily occurs in patients with immunodeficiencies, but novel risk factors continue to emerge, given the growing immunocompromised patient population. Human immunodeficiency virus (HIV) remains the primary risk factor in global series, though the introduction of antiretroviral therapy has led to a significant decrease in the incidence of cryptococcosis among persons with HIV [1]. The number of cases in patients without HIV who have undergone organ or hematopoietic cell transplant continues to increase. More cases are also being reported among non-HIV, non-transplant (NHNT) patients due to malignancies, autoimmune diseases, diabetes mellitus, renal dysfunction, hepatic cirrhosis, or the receipt of immunosuppressive medications [2,3,4].

Recently, reports of coronavirus disease-associated pulmonary aspergillosis (CAPA) [5,6] and COVID-19-associated mucormycosis (CAM) [7] have been extensively described. Though the association between COVID-19 and cryptococcosis is unknown, cases of cryptococcosis in patients with COVID-19 have been reported [8]. Initial reports suggested mortality rates greater than 60%, in which some cases of cryptococcosis were identified postmortem [8]. Data from a multicenter research network found that cryptococcosis occurred most often in hospitalized patients with COVID-19 who had traditional risk factors with an incidence and prevalence of 0.022% and 0.059%, respectively [9]. More cases were identified in patients who were elderly, male, identified as Hispanic or Latino, and required admission to the intensive care unit (ICU) for management of COVID-19. Overall, mortality was 36% among patients with cryptococcosis following COVID-19, which was significantly higher compared to patients without cryptococcosis. As such, the clinical characteristics, risk factors, and complications of cryptococcosis may be different among patients with and without COVID-19. Therefore, a greater understanding of the epidemiology and outcomes of cryptococcosis in patients with and without COVID-19 is needed.

## 2. Methods

The objectives were to compare demographic and laboratory characteristics, sites of infection, and outcomes of cryptococcosis between patients with COVID-19 to non-COVID-19 controls.

### 2.1. Study Population

Patients 18 years and older with cryptococcosis were identified from TriNetX on 11 July 2022 (Supplementary Materials). TriNetX is a global federated research network, which undergoes rigorous quality assessment to ensure data accuracy and cleanliness [10]. Cryptococcosis was defined by International Classification of Diseases, Tenth Revision, Clinical Modification (ICD-10-CM) diagnosis codes (Supplementary Materials).

The patient population was divided into two cohorts based on a diagnosis of COVID-19 within 3 months of the index diagnosis of cryptococcosis. The earliest encounter for cryptococcosis was identified as the index date in patients with multiple encounters. COVID-19 within 3 months of the index diagnosis of cryptococcosis (cases) was identified by logical observation identifiers names and codes (LOINCs) for SARS-CoV-2 or ICD-10-CM diagnosis codes for COVID-19. Controls were defined as patients with cryptococcosis but without COVID-19. A subgroup analysis was performed for cases and controls without HIV.

Demographics, comorbidities, medications, laboratory data, and outcomes were analyzed. Demographic data included age at the index event, sex, race, and ethnicity, as entered into TriNetX from electronic medical records. ICD-10-CM diagnosis codes were used to identify comorbidities during the 30 days prior to the diagnosis of cryptococcosis (Table S1). Comorbidities included previously identified risk factors for cryptococcosis (e.g., HIV, transplant, neoplastic diseases, hepatic fibrosis and cirrhosis, type 2 diabetes mellitus [DM2], chronic kidney disease [CKD], and autoimmune and inflammatory diseases) [11,12] and COVID-19 (e.g., HIV, transplant, neoplastic diseases, hepatic fibrosis and cirrhosis, DM2, CKD, autoimmune and inflammatory diseases, and heart failure) [13]. Laboratory data were the most recent labs between the index event and 30 days prior (Table S2). Immunosuppressive medication use was characterized by receipt of immune suppressants or immunomodulatory therapies (Table S3). Outcomes included ED visit, hospitalization, ICU admission, mechanical ventilation, and death (Table S4). Records in TriNetX are checked against a list of required fields (e.g., patient identifier) and rejects those records for which the required information is missing [10]. No imputations were made for missing comorbidity, medication, or laboratory data.

### 2.2. Outcome Measures

The primary outcome was the proportion of deaths in each group. The secondary outcomes studied were the proportion of patients in each group with underlying comorbidities, receiving immunosuppressive medications, requiring an ED visit, hospitalization, ICU admission, or mechanical ventilation.

### 2.3. Statistical Analysis

The TriNetX platform was used to complete statistical analyses. Descriptive statistics were presented as means and standard deviations for continuous variables, and as frequency and proportions for categorical variables. Continuous data were compared with independent *t*-tests, whereas categorical data were compared with χ^2^ or Fisher’s exact test, as appropriate. Outcomes were reported before and after propensity score matching (PSM). PSM was performed to control for differences between groups based on established risk factors for development of and mortality from cryptococcosis, including age, male sex, Hispanic or Latino ethnicity, HIV, transplant, or neoplastic disease, using 1:1 greedy nearest-neighbor algorithms [14]. We calculated odds ratios (ORs) with 95% confidence intervals (CIs) for immunosuppressive medications, ED visits, hospitalization, ICU admission, mechanical ventilation, and mortality, with *p* < 0.05 as the cut off for statistical significance.

## 3. Results

### 3.1. Demographic Characteristics

Among the 6998 patients with cryptococcosis included in the study, 4.4% (*n* = 306) had COVID-19 prior to cryptococcosis. Most patients in both groups were male, but those with COVID-19 were significantly older (Table 1). In both groups, more than 50% identified as White patients and 25% identified as Black or African American patients. While few Hispanic or Latino patients were in either group, patients with COVID-19 were more likely to be non-Hispanic. After PSM, 296 patients remained in each cohort with similar demographic characteristics.

### 3.2. Comorbidity- and Medication-Related Risk Factors

A higher proportion of patients with COVID-19 had a history of transplant or neoplastic disease. Approximately 30% of patients from each group had HIV. While less common in both groups, more patients with COVID-19 also had autoimmune diseases, such as sarcoidosis and rheumatoid arthritis. Additionally, a greater proportion of patients with COVID-19 had immunodeficiencies due to drugs (8% vs. 1%, *p* < 0.001) or an unspecified reason (18% vs. 4%, *p* < 0.001). Chronic comorbidities, including DM2, heart failure, and CKD were more common among those with a history of COVID-19. Higher rates of neoplastic diseases, heart failure, and CKD persisted among patients with COVID-19 after PSM.

Mean leukocyte, lymphocyte, and CD4 cell counts were not significantly different between groups. Notably, the mean CD4 cell count was less than 200 cells/μL in both groups. Despite higher proportions of CKD and DM2 in patients with COVID-19, serum creatinine and A1C were similar when compared to those without COVID-19. Acute phase reactants, including ferritin and LDH, but not CRP, were higher in patients with COVID-19. However, after PSM, laboratory values were similar between groups.

**Figure 1:**
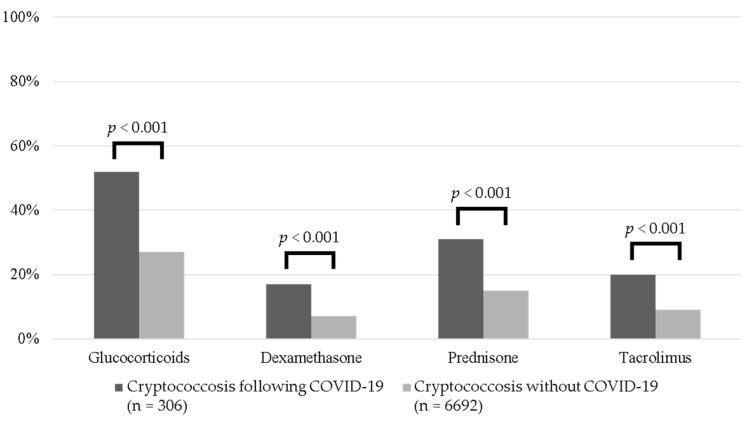
Proportion of patients who received immunosuppressive therapies among those with cryptococcosis following COVID-19 compared to non-COVID-19 controls with cryptococcosis.

Immunosuppressive medications were more common among patients with COVID-19 compared to non-COVID-19 controls. Glucocorticoids, including both prednisone and dexamethasone, and tacrolimus were approximately two times higher in patients with COVID-19 (Figure 1). However, other biological and small molecule targeted immunomodulatory therapies were uncommon in both groups. Following PSM, patients with COVID-19 remained at increased odds of receiving glucocorticoids (OR 1.73, 95% CI 1.25, 2.39, *p* < 0.001) and dexamethasone (OR 2.35, 95% CI 1.41, 3.95, *p* = 0.001) versus those without COVID-19.

### 3.3. Sites of Infection

**Figure 2:**
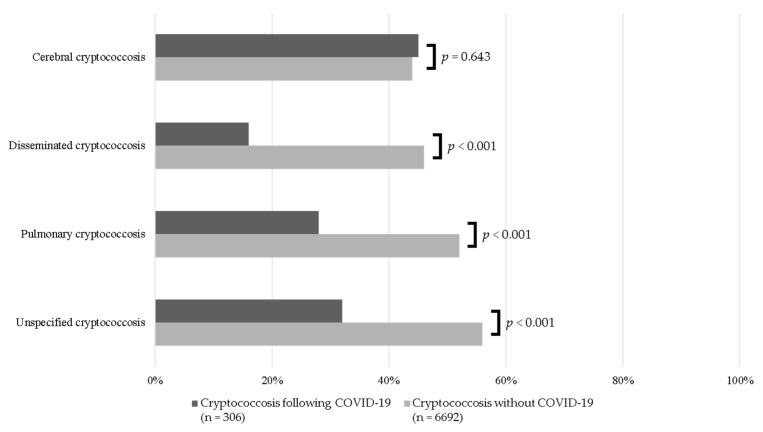
Types of cryptococcosis among patients with a history of COVID-19 compared to non-COVID-19 controls.

The types of cryptococcosis significantly differed between patients with and without COVID-19 (Figure 2). Fewer patients with COVID-19 had pulmonary, disseminated, or unspecified cryptococcosis compared to non-COVID-19 controls. However, cerebral cryptococcosis was more common among those with a history of COVID-19. Cutaneous, osseous, and other forms of cryptococcosis occurred in less than 3% of patients from either group.

### 3.4. Outcomes

The overall mortality was 11%, which was significantly higher among patients with COVID-19 (Table 2). While more than 50% of patients in each group required hospitalization, a significantly higher proportion of patients with COVID-19 required ED visits and ICU admission. After PSM, patients with COVID-19 had similar outcomes compared to non-COVID-19 controls.

### 3.5. Subgroup Analysis of Cases and Controls without HIV

Among the 4433 patients with cryptococcosis without HIV, those with COVID-19 were older than the non-COVID-19 controls (59.0 ± 13.7 years vs. 56.6 ± 14.9 years, *p* = 0.030). Both groups were comprised of a higher proportion of patients who were male (62% vs. 63%, *p* = 0.673) and identified as White (65% vs. 65%, *p* = 0.884), while more patients with COVID-19 identified as Hispanic or Latino (21% vs. 11%, *p* < 0.001). More patients with COVID-19 had a history of transplant (44% vs. 19%, *p* < 0.001), neoplastic disease (32% vs. 25%, *p* = 0.049), and unspecified immunodeficiency (25% vs. 5%, *p* < 0.001), as well as chronic comorbidities, including DM2 (42% vs. 25%, *p* < 0.001), heart failure (22% vs. 11%, *p* < 0.001), and CKD (49% vs. 23%, *p* < 0.001). Laboratory data were similar between groups, except for serum creatinine, which was higher among patients with COVID-19 (2.1 ± 2.3 mg/dL vs. 1.5 ± 1.5 mg/dL, *p* < 0.001).

More patients with COVID-19 received glucocorticoids (59% vs. 32%, *p* < 0.001), including both dexamethasone (21% vs. 8%, *p* < 0.001) and prednisone (40% vs. 20%, *p* < 0.001), as well as tacrolimus (31% vs. 13%, *p* < 0.001), while other immunosuppressive therapies were uncommon in both groups. After PSM, higher serum creatinine (2.1 ± 2.3 mg/dL vs. 1.4 ± 1.0 mg/dL, *p* = 0.001) and a history of CKD (47% vs. 36%, *p* = 0.010), DM2 (41% vs. 27%, *p* = 0.005) or dexamethasone use (22% vs. 10%, *p* = 0.001) persisted in patients with COVID-19.

Compared to non-COVID-19 controls, those with COVID-19 had lower proportions of pulmonary (35% vs. 55%, *p* < 0.001), cerebral (31% vs. 34%, *p* = 0.324), and disseminated cryptococcosis (18% vs. 44%, *p* < 0.001). However, unspecified cryptococcosis accounted for 39% of cases among patients with COVID-19 and 56% of non-COVID-19 controls (*p* < 0.001).

Hospitalization occurred in 54% of patients from each group (*p* = 0.906) but more patients with COVID-19 required ICU admission (17% vs. 12%, *p* = 0.015). However, mortality was similar between groups (16% vs. 13%, *p* = 0.219). Significant differences in ED visits, hospitalization, ICU admission, mechanical ventilation, and death were not observed after PSM.

## 4. Discussion

Cryptococcosis primarily affects patients with cell-mediated immunodeficiencies, although the populations at greatest risk have significantly changed over the last 50 years [1,2,3,4]. Other underlying comorbidities and conditions may be recognized as independent or additional risk factors for cryptococcosis as the number of cases in NHNT patients continue to increase. Reports of cryptococcosis following COVID-19 have been published [8], but the effect of COVID-19 on the risk and prognosis of cryptococcosis is incompletely understood. We used a global health research network to evaluate the epidemiology of cryptococcosis among patients with and without COVID-19. Overall, 4.4% of all patients had COVID-19 within 3 months of the index diagnosis of cryptococcosis with a mortality rate of 14%. In our previous study, we identified 65 patients who developed cryptococcosis among 212,479 patients hospitalized with COVID-19 [9]. Those with cryptococcosis following COVID-19 were significantly more likely to have a history of HIV, transplant, hepatic cirrhosis, DM2, or treatment with tocilizumab or baricitinib compared to patients with COVID-19 but without cryptococcosis. In addition, worse outcomes, including increased ICU requirements, mechanical ventilation, and mortality, were observed in patients with cryptococcosis following COVID-19.

Pulmonary and cerebral cryptococcosis were most common among both groups in our study, but the proportions of each type varied between patients with and without COVID-19. Despite meningoencephalitis being the most common life-threatening complication of cryptococcosis, cerebral cryptococcosis was diagnosed in 41% of all patients, which was more common among those with a history of COVID-19. Cerebral cryptococcosis was more common among patients with HIV with a higher proportion observed in those with COVID-19 (87% [*n* = 80/92] vs. 71% [*n* = 1379/1954]). The overall distribution of cerebral cryptococcosis was lower while pulmonary cryptococcosis was higher compared to previous findings in non-COVID-19 patients (44% vs. range 48–61% and 51% vs. range 24–34%, respectively) [2,3,4,15]. These differences may be attributed to the large proportion of patients in our study categorized as unspecified and other types of cryptococcosis based on ICD-10-CM diagnosis codes, but previous studies included patients from 1996 to 2017 [2,3,4,15].

Few differences in demographic characteristics were noted between patients with and without COVID-19 but were comparable to data published prior to the COVID-19 pandemic [2,3,4,15]. The majority of patients with cryptococcosis from prior studies were male (range 60–85%) between 40 and 60 years [2,3,4,15]. In contrast, patients with HIV were younger (range 40–43 years) and more often identified as Black or African American compared to those with a history of transplant or NHNT patients [2,3,15]. Patients in our study were of similar age but those with HIV were older than those from previous cohorts [3,15,16]. Additionally, most patients in our study identified as White, regardless of HIV status. Though the differences in age and ethnicity were significant between groups in our study, the findings are most likely due to the distribution of COVID-19 in the U.S., particularly during the early stages of the pandemic [17].

Patients with COVID-19 experienced a greater burden of comorbidities compared to non-COVID-19 controls. Approximately 30% of patients had HIV, which was evenly distributed between those with COVID-19 and non-COVID-19 controls. The proportion of patients with HIV in our study was substantially less than that reported in previous cohorts from 1997 to 2009 (79%) [18], 2000 to 2007 (64%) [19], and 2004 to 2012 (56%) [2]. Notably, the median CD4 cell count was greater than 100 cells/μL in both groups from our study, though this laboratory value was obtained in 7% of patients from each group. The low proportion of patients with HIV in our study is consistent with the gradual reduction in the incidence of cryptococcosis among persons with HIV in the U.S.

Alternatively, patients with a history of transplant or neoplastic disease comprised a large percentage of the patients included our study (13% and 21%, respectively) compared to previous data [2]. Significantly more patients with COVID-19 had a history of transplant or neoplastic disease compared to non-COVID-19 controls. However, a history of transplant or neoplastic disease was most common in patients with COVID-19 but no history of HIV (44% and 32%, respectively). Other comorbidities, including autoimmune and inflammatory diseases, DM2, heart failure, and CKD, some of which are risk factors for cryptococcosis, were also more common among patients with COVID-19 compared to non-COVID-19 controls. Similar findings were also identified for cases and controls without HIV. While transplant recipients and patients with malignancies or comorbidities are at increased risk for SARS-CoV-2 infection, including severe COVID-19, the relationship between COVID-19 and cryptococcosis remains poorly characterized.

Cases of disseminated cryptococcosis following influenza, either H1N1 or H7N9, have been reported in otherwise healthy patients with prolonged, severe influenza, some of whom received immunosuppressive therapies [20,21]. While the pathogenesis of and host responses to influenza and SARS-CoV-2 differ, both may elicit extensive pulmonary damage, dysregulated immune response, and severe lymphopenia [22]. Following the inhalation of *Cryptococcus neoformans*, patients with significant impaired immunity may develop acute pulmonary disease, whereas in others, *C*. *neoformans* may reside within phagocytic cells and potentially escape, leading to unregulated, widespread dissemination [23,24]. A previous study using human macrophages identified viral infections increased the vomocytosis, a nonlytic exocytosis from macrophages, of *C*. *neoformans* [25]. In the setting of SARS-CoV-2 infection, severely impaired cell-mediated immunity and hyperinflammation may influence the host’s ability to contain *C*. *neoformans*. In our previous study, cryptococcosis most often occurred within 10 days following the index hospitalization with COVID-19 [9]. While some cases may represent previously unrecognized cryptococcosis prior to COVID-19, acute pulmonary and disseminated cryptococcosis has been reported following treatment with short-term glucocorticoids [26]. Though additional data are needed, the pathogenesis of cryptococcosis following COVID-19 is complex and likely depends on the virulence of the respiratory virus combined with the host immune response, which may be influenced by comorbidities and immunosuppressive therapies [27,28,29].

The clinical benefit of Immunosuppressive therapies in many diseases, including COVID-19, is well established, but the collateral effects on the host immune response may increase the risk for secondary infections caused by opportunistic pathogens. Glucocorticoid use was significantly higher among patients with COVID-19 compared to non-COVID-19 controls. The increased use of prednisone and dexamethasone in patients with COVID-19 may reflect the higher percentage of patients with autoimmune diseases and management of COVID-19. While glucocorticoid doses and durations are unknown in our study, cases of cryptococcosis have been reported following both short- and long-term treatment with glucocorticoids [26]. Likewise, the increased receipt of tacrolimus likely represents the greater number of transplant recipients with COVID-19 in our data. Interestingly, the limited use of tocilizumab and baricitinib in the COVID-19 group raises important questions about whether patient-specific factors prohibited their use.

Outcomes in patients with cryptococcosis are dependent upon numerous factors, including the host response and extent of disease. In this study, we observed higher rates of ED visits, ICU admissions, and death in patients with COVID-19 prior to cryptococcosis compared to those without COVID-19. However, the significant differences in outcomes did not persist after PSM. Alternatively, previously published reports of cryptococcosis in patients with COVID-19 have demonstrated the high rates of ICU admission [30], mechanical ventilation [30], and mortality, ranging from 36% to 63% [8,9], potentially. The difference in these outcomes may be attributable to more severe cryptococcosis or COVID-19 or potential delays in the diagnosis or initiation of antifungal therapy. While the severity of COVID-19 was unknown in our study, these findings question whether SARS-CoV-2 infection may contribute to worse outcomes in patients with cryptococcosis.

Although we compared the clinical characteristics and outcomes of cryptococcosis in patients with and without COVID-19, our study was retrospective and utilized aggregate data from a multicenter research network that includes institutions across the U.S. However, findings from studies using large databases may provide greater generalizability given their “real world” outcomes and complement results obtained from single centers. Demographic characteristics, comorbidities, diagnoses, and outcomes derived from various codes may be inaccurate due to under coding or misclassification, but numerous mechanisms within TriNetX ensure high quality data [10]. Notably, previous studies demonstrated high positive predictive values for cryptococcosis using ICD-9-CM diagnosis codes [2,31]. Medication dosages, durations, and indications were not included in the TriNetX database. In addition, laboratory tests were not obtained for all patients, likely due to differences in institutional and clinical practices (e.g., A1C reported in 10% of patients). Lastly, relying on LOINCs for SARS-CoV-2 or ICD-10-CM diagnosis codes for COVID-19 may not account for patients with self-diagnosed or undiagnosed COVID-19 or individuals who were diagnosed with COVID-19 at an institution not affiliated with TriNetX. We were unable to account for the COVID-19 severity in the study, so were unable to isolate the subgroup of incidental SARS-CoV-2 infections or asymptomatic COVID-19 for study. However, this may have affected both cohorts.

Patients with cryptococcosis following COVID-19 had higher rates of comorbidities and glucocorticoid use compared to those with cryptococcosis without COVID-19. Despite this, those with COVID-19 did not require more ED visits or ICU admissions and were not at higher risk of death. Until the relationship between cryptococcosis and SARS-CoV-2 infection is better understood, a greater suspicion and recognition of cryptococcosis as a potential complication following COVID-19 is imperative for early diagnosis and treatment to improve patient outcomes.

## Figures and Tables

**Table 1:** Baseline characteristics of patients with cryptococcosis following COVID-19 compared to non-COVID-19 controls with cryptococcosis.

	Before Matching			After Matching		
Variable	Cryptococcosis Following COVID-19 (*n* = 306)	Cryptococcosis without COVID-19 (*n* = 6692)	*p* Value	Cryptococcosis Following COVID-19 (*n* = 296)	Cryptococcosis without COVID-19 (*n* = 296)	*p* Value
	Demographics					
Age at index event (years), mean (SD)	55.2 (14.4)	51.9 (15.2)	<0.001	55.1 (14.4)	55.3 (14.8)	0.890
Male sex	68 (208)	69 (4291)	0.705	68 (200)	68 (201)	0.930
White	175 (57)	3421 (55)	0.454	57 (169)	58 (172)	0.803
Black or African American	79 (26)	1586 (26)	0.917	26 (78)	23 (68)	0.640
Asian	10 (3)	102 (2)	0.032	3 (10)	3 (10)	1
Unknown race	44 (14)	1080 (17)	0.177	14 (41)	16 (48)	0.421
Hispanic or Latino	51 (17)	787 (13)	0.041	16 (48)	16 (47)	0.911
Non-Hispanic	218 (71)	3945 (63)	0.006	72 (212)	63 (186)	0.023
	Underlying comorbidities					
HIV	92 (30)	1954 (31)	0.618	31 (92)	32 (95)	0.791
Transplanted organs or tissues	89 (29)	806 (13)	<0.001	27 (80)	29 (86)	0.583
Neoplastic diseases	114 (37)	1329 (21)	<0.001	37 (109)	27 (80)	0.011
Immunodeficiency with predominantly antibody defects	10 (3)	69 (1)	0.001	3 (10)	3 (10)	1
Combined immunodeficiencies	0 (0)	10 (<1)	0.483	0 (0)	0 (0)	–
Common variable immunodeficiency	10 (3)	17 (<1)	<0.001	3 (10)	3 (10)	1
Other immunodeficiencies *	70 (23)	300 (5)	<0.001	21 (61)	20 (59)	0.838
Sarcoidosis	14 (5)	141 (2)	0.010	5 (14)	3 (10)	0.405
Systemic connective tissue disorders	19 (6)	256 (4)	0.075	6 (19)	5 (15)	0.480
Rheumatoid arthritis	10 (3)	19 (<1)	<0.001	3 (10)	3 (10)	1
Noninfective enteritis and colitis	30 (10)	428 (7)	0.051	10 (28)	11 (31)	0.681
Hepatic fibrosis and cirrhosis	19 (6)	396 (6)	0.912	6 (17)	7 (21)	0.502
Type 2 diabetes mellitus	106 (35)	1158 (19)	<0.001	34 (10)	28 (82)	0.109
Heart failure	62 (20)	514 (8)	<0.001	19 (57)	12 (35)	0.013
Chronic kidney disease	127 (42)	1096 (18)	<0.001	40 (119)	29 (86)	0.004
	Laboratory values					
Leukocytes (K/μL), mean (SD)	18.7 (147.5)	19.3 (213)	0.982	19 (149.6)	7.5 (6.6)	0.304
Lymphocytes (K/μL), mean (SD)	6.4 (22.3)	6.8 (41.8)	0.916	6.6 (22.7)	5.1 (36.8)	0.703
CD4 cells (cells/μL), mean (SD)	108 (141)	175 (306)	0.332	108 (141)	151 (243)	0.485
Serum creatinine (mg/dL), mean (SD)	1.7 (1.9)	1.5 (1.5)	0.033	1.7 (1.9)	1.6 (1.5)	0.554
Hemoglobin A1C (%), mean (SD)	6.9 (2.2)	6.5 (1.8)	0.110	6.9 (2.3)	7.0 (1.9)	0.849
Ferritin (ng/mL), mean (SD)	2240 (9887)	1004 (1815)	0.020	2296 (10,176)	617 (645)	0.476
C-reactive protein (mg/dL), mean (SD)	49.5 (62.7)	36.4 (57.9)	0.078	50.7 (63.2)	34.9 (51.3)	0.238
Lactate dehydrogenase (units/L), mean (SD)	551 (1034)	356 (497)	0.004	553 (1047)	292 (172)	0.113

Data are presented as n (%) unless otherwise noted. COVID-19, coronavirus disease 2019; SD, standard deviation. * other immunodeficiencies included lymphocyte function antigen-1 defects, complement system defects, as well as immunodeficiency due to other conditions, medications, external causes, or unspecified causes.

**Table 2:** Differences in outcomes among patients with cryptococcosis following COVID-19 compared to non-COVID-19 controls with cryptococcosis.

	Before Matching				After Matching			
Variable	Cryptococcosis Following COVID-19 (*n* = 306)	Cryptococcosis without COVID-19 (*n* = 6219)	OR (95% CI)	*p* Value	Cryptococcosis Following COVID-19 (*n* = 296)	Cryptococcosis without COVID-19 (*n* = 296)	OR (95% CI)	*p* Value
ED visit	88 (29)	1442 (23)	1.337 (1.037–1.725)	0.025	86 (29)	69 (23)	1.347 (0.932, 1.947)	0.112
Hospitalization	169 (55)	3529 (57)	0.940 (0.746, 1.184)	0.601	163 (55)	184 (62)	0.746 (0.537, 1.036)	0.080
ICU admission	56 (18)	678 (11)	1.8 (1.355, 2.472)	<0.001	54 (18)	43 (15)	1.313 (0.848, 2.034)	0.222
Mechanical ventilation	36 (12)	646 (10)	1.150 (0.805, 1.644)	0.442	35 (12)	30 (10)	1.189 (0.709, 1.993)	0.511
Death	44 (14)	653 (11)	1.431 (1.030, 1.990)	0.032	39 (13)	34 (12)	1.169 (0.76, 1.911)	0.532

Data are presented as n (%) unless otherwise noted. COVID-19, coronavirus disease 2019; CI, confidence interval; ED, emergency department; ICU, intensive care unit; OR, odds ratio.

## Data Availability

The data that support the findings of this study are available from TriNetX. Restrictions apply to the availability of these data, which were used under license for this study. Data are available from TriNetX through a third-party agreement option.

## Supplementary Materials

The following supporting information can be downloaded at: https://www.mdpi.com/article/10.3390/jof8111234/s1, details regarding TriNetX [9,32,33,34,35], composition of the study population, Table S1: International Classification of Diseases, Tenth Revision, Clinical Modification (ICD-10-CM) diagnosis codes for underlying comorbidities; Table S2: Logical Observation Identifiers Names and Codes (LOINC) for laboratory values; Table S3: RxNorm codes for medications; Table S4: Outcomes based on Current Procedural Terminology (CPT) codes, HL7 Terminology, and International Classification of Diseases, Ninth Revision or Tenth Revision, Clinical Modification (ICD-9-CM or ICD-10-CM) diagnosis codes.
