# Supplementary material for: Characteristics and Outcomes of Cryptococcosis among Patients with and without COVID-19
Source: J Fungi (Basel). 2022 Nov 21;8(11):1234. doi: 10.3390/jof8111234 (PMC9695249; doi:10.3390/jof8111234)
Supplement: Supplementary file 1 [file jof-08-01234-s001.zip › jof-2020375-supplementary.pdf]

SUPPLEMENTARY MATERIALS

TriNetX Network

The following details are based on our previous description of the TriNetX Network [1]

The data used in this study was collected from the TriNetX Network, which is a global federated health research network providing access to electronic medical records across healthcare organizations, including hospitals, primary care, and specialist providers, from more than 80 million insured and uninsured patients. Available data include demographic characteristics, based on International Classification of Diseases, Tenth Revision, Clinical Modification (ICD-10-CM) diagnosis codes, procedures, medications, and measurements (e.g., laboratory test results). Data are continuously updated at various times. Cohorts can be created based on specified inclusion and exclusion criteria and matched for confounding variables using the built-in propensity score matching capability on the TriNetX platform. Outcomes can then be compared between cohorts over defined time periods. TriNetX, LLC is compliant with the Health Insurance Portability and Accountability Act (HIPAA), the US federal law which protects the privacy and security of healthcare data, and any additional data privacy regulations applicable to the contributing healthcare organizations. TriNetX is certified to the ISO 27001:2013 standard and maintains an Information Security Management System (ISMS) to ensure the protection of the healthcare data it has access to and to meet the requirements of the HIPAA Security Rule. Any data displayed on the TriNetX Platform in aggregate form, or any patient level data provided in a data set generated by the TriNetX Platform, only contains de-identified data as per the de-identification standard defined in Section §164.514(a) of the HIPAA

Privacy Rule. The process by which the data is de-identified is attested to through a formal determination by a qualified expert as defined in Section §164.514(b)(1) of the HIPAA Privacy Rule.

Further details regarding TriNetX are available in previously published literature [2–5]

Study population

- Patient identification

Patients 18 years and older with cryptococcosis were identified by at least one of the following: B45 (Cryptococcosis), B45.0 (Pulmonary cryptococcosis), B45.1 (Cerebral cryptococcosis), B45.2 (Cutaneous cryptococcosis), B45.3 (Osseous cryptococcosis), B45.7 (Disseminated cryptococcosis), B45.8 (Other forms of cryptococcosis), or B45.9 (Unspecified cryptococcosis).

- Case definition

Must have the following within 3 months prior to or on the same date as the index diagnosis of cryptococcosis: J12.82 (Pneumonia due to COVID-19) OR U07.1 (COVID-19) OR U07.2 (COVID-19) OR 94558-4 (SARS coronavirus 2 Ag [Presence] in Respiratory specimen by Rapid immunoassay [Positive]) OR 94558-4 (SARS coronavirus 2 Ag [Presence] in Respiratory specimen by Rapid immunoassay [Positive]) OR 9088 (SARS coronavirus 2 and related RNA [Presence] [Positive]) OR 96119-3 (SARS-CoV-2 (COVID-19) Ag [Presence] in Upper respiratory specimen by Immunoassay [Positive]) OR 95209-3 (SARS coronavirus+SARS coronavirus 2 Ag [Presence] in Respiratory specimen by Rapid immunoassay [Positive]) OR 97097-0 (SARS-CoV-2 (COVID-19) Ag [Presence] in Upper respiratory specimen by Rapid immunoassay [Positive]).

- Control definition

Cannot have the following: J12.82 (Pneumonia due to COVID-19) OR U07.1 (COVID-19) OR U07.2 (COVID-19) OR 94558-4 (SARS coronavirus 2 Ag [Presence] in Respiratory specimen by Rapid immunoassay [Positive]) OR 94558-4 (SARS coronavirus 2 Ag [Presence] in Respiratory specimen by Rapid immunoassay [Positive]) OR 9088 (SARS coronavirus 2 and related RNA [Presence] [Positive]) OR 96119-3 (SARS-CoV-2 (COVID-19) Ag [Presence] in Upper respiratory specimen by Immunoassay [Positive]) OR 95209-3 (SARS coronavirus+SARS coronavirus 2 Ag [Presence] in Respiratory specimen by Rapid immunoassay [Positive]) OR 97097-0 (SARS-CoV-2 (COVID-19) Ag [Presence] in Upper respiratory specimen by Rapid immunoassay [Positive]).

- Subgroup analysis

A subgroup analysis was performed for cases and controls with no history of HIV using International Classification of Diseases, Tenth Revision, Clinical Modification (ICD-10-CM) diagnosis codes for HIV (cannot have B20).

Supplementary Table S1. International Classification of Diseases, Tenth Revision, Clinical Modification (ICD-10-CM) diagnosis codes for underlying comorbidities

Code	Description
B20	HIV infection
C00-D49	Neoplasm
D80	Immunodeficiency with predominantly antibody defects
D81	Combined immunodeficiencies
D83	Common variable immunodeficiency
D84	Other immunodeficiencies
D86	Sarcoidosis
E11	Diabetes mellitus
I50	Heart failure
K50-K52	Noninfective enteritis and colitis
K74	Hepatic fibrosis and cirrhosis
M05	Rheumatoid arthritis
M30-M36	Systemic connective tissue disorders
N18	Chronic kidney disease
Z94	Transplanted organs or tissues

Supplementary Table S2. Logical Observation Identifiers Names and Codes (LOINC) for laboratory values

Code	Description
9015	Leukocytes
731-0	Lymphocytes
24467	CD4 cells
9037	Hemoglobin a1c
9048	Aspartate aminotransferase
9044	Alanine aminotransferase
9046	Alkaline phosphatase
9045	Albumin
9024	Serum creatinine
9042	Ferritin
9063	C reactive protein
9052	Lactate dehydrogenase

Supplementary Table S3. RxNorm codes for medications

Code	Description
HS051	Glucocorticoids
3264	Dexamethasone
8640	Prednisone
612865	Tocilizumab
2047232	Baricitinib
42316	Tacrolimus

Supplementary Table S4. Outcomes based on Current Procedural Terminology (CPT) codes, HL7 Terminology, and International Classification of Diseases, Ninth Revision or Tenth Revision, Clinical Modification (ICD-9-CM or ICD-10-CM) diagnosis codes

Code(s)	Description
99281, 99282, 99283, 99284, 99285<br>HL7V3.0:VisitType:EMER	ED visit
1013659, 1013660, 1013699, 1013729<br>HL7V3.0:VisitType:IMP, HL7V3.0:VisitType:NONAC, HL7V3.0:VisitType:SS	Hospitalization
1013729, 1014309, 99291	Critical care services
1015098, 31500, 1022227<br>5A1905Z, 5A1935Z, 5A1945Z, 5A1955Z, 0BH17EZ, 0BH18EZ, 0BH13EZ, 39.65†	Mechanical ventilation
†, denotes an ICD-9 CM code as CMS has not released mapping for new ICD-10-CM/PCS codes	

References

1. Chastain, D.B.; Kung, V.M.; Golpayegany, S.; Jackson, B.T.; Franco-Paredes, C.; Vargas Barahona, L.; Thompson, G.R., 3rd; Henao-Martínez, A.F. Cryptococcosis among hospitalised patients with COVID-19: A multicentre research network study. *Mycoses* **2022**, *65*, 815–823. https://doi.org/10.1111/myc.13476.

2 Taquet, M.; Luciano, S.; Geddes, J.R.; Harrison, P.J. Bidirectional associations between COVID-19 and psychiatric disorder: retrospective cohort studies of 62 354 COVID-19 cases in the USA. *Lancet Psychiatry* **2021**, *8*, 130–140. https://doi.org/10.1016/S2215-0366(20)30462-4.

3. Taquet, M.; Sillett, R.; Zhu, L.; Mendel, J.; Camplisson, I.; Dercon, Q.; Harrison, P.J. Neurological and psychiatric risk trajectories after SARS-CoV-2 infection: an analysis of 2-year retrospective cohort studies including 1 284 437 patients. *Lancet Psychiatry* **2022**, *9*, 815–827. https://doi.org/10.1016/S2215-0366(22)00260-7.

4. Tang, K.; Seo, J.; Tiu, B.C.; Le, T.K.; Pahalyants, V.; Raval, N.S.; Semenov, Y.R.; Ugwu-Dike, P.O.; Zubiri, L.; Vivek N.; et al. Association of Cutaneous Immune-Related Adverse Events With Increased Survival in Patients Treated With Anti-Programmed Cell Death 1 and Anti-Programmed Cell Death Ligand 1 Therapy. *JAMA Dermatol*. **2022**, *158*, 189–193. https://doi.org/10.1001/jamadermatol.2021.5476.

5. Mandadi, S, Pulluru, H, Annie, F. Comparative outcomes of combined corticosteroid and remdesivir therapy with corticosteroid monotherapy in ventilated COVID-19 patients. *PLoS ONE* **2022**, *17*, e0264301. https://doi.org/10.1371/journal.pone.0264301.
